# Chronic progressive HIV-1 infection is associated with elevated levels of myeloid-derived suppressor cells

**DOI:** 10.1186/1742-4690-9-S2-P283

**Published:** 2012-09-13

**Authors:** T Vollbrecht, J Roider, R Stirner, A Tufman, RM Huber, JR Bogner, A Lechner, C Bourquin, R Draenert

**Affiliations:** 1 Ludwig-Maximilians-University, Munich, Germany; 2 University of Fribourg, Fribourg, Switzerland

## Background

Myeloid-derived suppressor cells (MDSC) have been described as suppressors of T cell functions in many tumor models. However MDSC in HIV-1 infection have not been studied to date. As impaired T cell function is a hallmark of chronic progressive HIV-1 infection, we hypothesized that MDSC also play a role here.

## Methods

Surface staining and FACS analysis were performed on freshly isolated PBMC of HIV-infected individuals and compared to healthy controls and individuals with lung carcinoma. MDSC of late-stage HIV-infected subjects were isolated using magnetic beads and co-cultured with the respective CD8 T cells for evaluation of proliferative capacity.

## Results

We found that chronically HIV-infected HAART-naïve individuals had significantly higher CD11b+CD14-CD33+CD15+ MDSC levels than healthy controls (p=0.01). MDSC frequencies showed a positive correlation with viral load (r2=0.24, p=0.0002) and a negative correlation with CD4 count (r2=0.29, p<0.0001). Initiation of HAART led to a rapid drop in MDSC levels. MDSC from HIV-infected progressors restricted the proliferative capacity of CD8 T cells from healthy donors and of Gag/Nef-specific CD8 T cells from HIV-controllers in vitro. Furthermore CD11b+CD14-CD33+CD15+ MDSC induced the expansion of CD4+CD25+FoxP3+ regulatory T cells when co-incubated with PBMC from controllers in vitro.

## Conclusion

We conclude that chronic uncontrolled HIV-infection is associated with elevated levels of MDSC which potentially contribute to the impaired T cell responses characteristic for the progressive disease stage.

